# Evaluating short-read sequence data from the highly redundant, novel transcriptome of *Polarella glacialis*

**DOI:** 10.1186/gb-2011-12-s1-p5

**Published:** 2011-09-19

**Authors:** Theodore R Gibbons, Gregory T Concepcion, Tsvetan R Bachvaroff, Charles F Delwiche

**Affiliations:** 1 Department of Cell Biology and Molecular Genetics, University of Maryland, College Park, MD 20742, USA; 2 Smithsonian Environmental Research Center, Edgewater, MD 21037, USA

## Background

Dinoflagellates are a diverse group of ecologically important eukaryotic algae, the global impact of which ranges from the large-scale primary production of oxygen [1] to devastating toxic algal blooms [2]. These organisms have exceptionally large genomes (10^9^ to 10^11^ bases) [3] and highly duplicated genes (which can occur thousands of times within a single genome) [4]. These and other unusual characteristics have made dinoflagellates difficult to study using traditional molecular biology techniques. Sequence data for dinoflagellates are correspondingly sparse, and not a single genome sequence has been published to date.

As part of our project called Assembling the Dinoflagellate Tree of Life (DAToL), our laboratory has sequenced the transcriptome of *Polarella glacialis*. Its genome is estimated to be only 3 Gb in size, making it one of the smallest known dinoflagellate genomes. Because we had to rely on *de novo* assemblers that had been tested using data from organisms that are extremely divergent from dinoflagellates, we took special care in our attempts to validate the data. Before expanding our analyses to include additional dinoflagellates, we compared the results from different sequencing and assembly methods.

## Methods

Total RNA was extracted from cultured *P. glacialis*. This sample was then divided and shipped to Macrogen for rRNA degradation, library preparation and sequencing. One library was sequenced on one-eighth of a Roche/454 GS FLX picotiter plate using Titanium chemistry. A second library was sequenced using one lane on an Illumina GAIIx sequencer for 78 cycles in both directions (paired end). The sequences were assembled using Newbler, MIRA, Oases and Trinity, and they were analyzed using various custom scripts.

## Results

The total amount of unassembled 454 sequence data added to less than one-third of the combined lengths of only those Trinity transcripts that had a significant BLAST hit against a sequence in GenBank, indicating that we did not achieve complete coverage with our 454 data.

## Conclusions

Our primary hypothesis was that the longer read lengths of the 454 data might allow the corresponding assemblers to better resolve repetitive sequences, which could be instrumental for assembling conserved regions within highly duplicated genes. Our failure to obtain complete coverage with the 454 dataset undermined our ability to test this hypothesis, although we made several other interesting observations. Notably, despite the vast disparity in the depth of the coverage between the 454 and Illumina assemblies, we observed unique, apparently real sequences within some of the 454 contigs.
